# Stress Strengthens Memory of First Impressions of Others' Positive Personality Traits

**DOI:** 10.1371/journal.pone.0016389

**Published:** 2011-01-26

**Authors:** Johanna Lass-Hennemann, Linn K. Kuehl, André Schulz, Melly S. Oitzl, Hartmut Schachinger

**Affiliations:** 1 Institute of Psychobiology, University of Trier, Trier, Germany; 2 Leiden/Amsterdam Center for Drug Research, Leiden, The Netherlands; 3 Institute of Psychobiology, University of Trier, Trier, Germany; University of Groningen, The Netherlands

## Abstract

Encounters with strangers bear potential for social conflict and stress, but also allow the formation of alliances. First impressions of other people play a critical role in the formation of alliances, since they provide a learned base to infer the other's future social attitude. Stress can facilitate emotional memories but it is unknown whether stress strengthens our memory for newly acquired impressions of other people's personality traits. To answer this question, we subjected 60 students (37 females, 23 males) to an impression-formation task, viewing portraits together with brief positive vs. negative behavior descriptions, followed by a 3-min cold pressor stress test or a non-stressful control procedure. The next day, novel and old portraits were paired with single trait adjectives, the old portraits with a trait adjective matching the previous day's behavior description. After a filler task, portraits were presented again and subjects were asked to recall the trait adjective. Cued recall was higher for old (previously implied) than the novel portraits' trait adjectives, indicating validity of the applied test procedures. Overall, recall rate of implied trait adjectives did not differ between the stress and the control group. However, while the control group showed a better memory performance for others' implied negative personality traits, the stress group showed enhanced recall for others' implied positive personality traits. This result indicates that post-learning stress affects consolidation of first impressions in a valence-specific manner. We propose that the stress-induced strengthening of memory of others' positive traits forms an important cue for the formation of alliances in stressful conditions.

## Introduction

People are social beings who readily form impressions of other people. We tend to evaluate others very rapidly on the basis of physical features and behavioral information [1], [2]. Most of the time we do not form impressions of others intentionally, but rather spontaneously and with minimal cognitive effort: We watch a person behave in some way towards ourselves or another person and infer personality traits from this behavioral information. Impression formation reflects some kind of social learning: During impression formation trait knowledge about a person is gained and this knowledge influences subsequent cognitions and/or responses regarding that person [3].

Peoples' daily lives are full of social encounters in which they form impressions of other people and some of those (for example job interviews) are experienced as stressful events. Many studies have investigated the effects of stress on learning and memory processes showing that stress administered shortly after learning (post-learning stress) facilitates memory consolidation [for a review see 4], [5]. However, the effects of stress for social relevant learning like the memory for our first impressions of other people have not been studied yet.

There is growing evidence for the notion that human social impression formation relies on a distinct set of cognitive processes, and that the cognitive processing of socially-relevant characteristics of other people differs from other kinds of thoughts. Three lines of research contribute to this notion:

First, behavioral studies of social cognition demonstrated that tasks with emphasis on the social relevant aspects of items produce a memory performance that differs from non-social tasks, both qualitatively (e.g., recall that clusters around spontaneous inferred personality traits) and quantitatively (e.g., better memory performance) [6], [7], [8], [9], [10].

Second, research with preverbal infants showed that the ability to evaluate others due to their behavior (e.g. form impressions of others) is already present in infants as young as 6 months [11], [12]. This lead Hamlin and colleagues [11] to the conclusion that the capacity to evaluate others may be innate and universal.

Third, functional imaging studies found an activation of a distinct region of the dorsal medial prefrontal cortex during impression formation compared to non-social tasks. Furthermore the degree of activation in the dorsal medial prefrontal cortex correlated with memory performance for the impressions, while activity the hippocampus correlated with memory for the non-social items [13], [14]. Moreover patients with hippocampal lesions show normal impression formation indicating that the hippocampus, which is thought to play a major role in declarative rather than implicit memory formation, is not critical for forming impressions of other people [15].

When people experience stress the body reacts to the stressor in a specific way. Stressful experiences lead to an activation of two biological stress systems: the hypothalamo-pituitary-adrenal axis and the autonomic sympathetic system. The activation of these two systems leads through intermediate steps to the release of glucocorticoids (in humans cortisol) and adrenalin (epinephrine) from the adrenal glands. The glucocorticoids directly reach the brain via the bloodstream and stimulate receptors in different brain regions, especially the hypothalamus, hippocampus, prefrontal cortex and the amygdala. Circulating adrenaline may not cross the blood brain barrier, but can indirectly (via vagal afferents and neurons of the solitary tract nucleus) induce the release of noradrenaline in the amygdala. This stress-induced amygdala activation affects memory consolidation by influencing neuroplasticity in other brain regions including (but not restricted to) the hippocampus. Besides the interaction between the glucocorticoid system and the adrenaline system the endocannabinoid system has also been proposed to mediate glucocorticoid effects on memory processes [16].

Whereas stress effects on hippocampus dependent memory were studied quiet often, this is not the case for impression formation. Impression formation is suggested to be under the regulation of the medial prefrontal cortex [13], [17]. This area is closely connected to the amygdala [18], [19] and is also a main target structure of stress-glucocorticoids [20]. Therefore it may be expected that post-learning stress has similar effects on prefrontal cortex dependent social relevant memory as on hippocampus-dependent declarative memory.

Spontaneous personality trait inferences derived from observed behaviors of others are thought to be a central part of peoples' impressions of others and have been frequently used to study spontaneous impression-formation [1], [3], [21]. To create initial personality impressions in our participants, we adopted a paradigm developed by Carlston and Skowronski [3]. In this paradigm participants first familiarize themselves with a series of portraits of people (“actors”) paired with personality trait-implying paragraphs that describe the actor's behavior. This task allows subjects to make personality trait inferences and to associate these with the person on the corresponding portrait. Then after intervals up to one week, they are instructed to learn pairs of portraits and trait adjectives without any reference to their prior familiarization experience. These portrait-trait adjective pairs include the actors' portraits presented earlier paired with the trait adjectives implied by the paragraphs with which they appeared. These “old-portrait-implied trait adjective” pairs are easier for participants to learn than new portrait-trait adjective combinations, because participants are asked to relearn something they already knew. The difference between learning performance for “old portrait-implied trait adjective” pairs and new pairs serves as an index of impression formation.

Here, we investigate whether and how post learning stress influences this form of implicit impression formation. Participants passed through a modified version of the Carlston and Skowronski paradigm with a post-learning stressor or a control procedure after the exposure to portraits of students combined with trait-implying paragraphs. Impression formation for these portrait-trait pairs was tested on the next day. We asked whether post-learning stress may affect the consolidation of first impressions, and whether such an effect would depend on the affective valence of the implied trait.

## Methods

### Participants

Participants were 60 students (37 women and 23 men, mean age 23.0 years, *SD* = 3.3, from the University of Trier, Germany), who responded to an advertisement offering 20 Euros for taking part in an experiment on emotional and physiological reactions to faces and pain. Participation was restricted to healthy non-smokers with a body mass index in the normal range of between 20 and 25 kg/m^2^, determined by a telephone screening interview that the respondent completed before being invited to take part. Physical exercise, alcohol, caffeinated drinks, and meals were not allowed 3 hours prior to each of the two experimental sessions. Experiments took place between 2 p.m. and 5 p.m. All participants gave their written informed consent. The research was approved by the ethical committee of the medical association of Rhineland-Palatinate.

### Materials Development and Pretesting

#### Portraits

The models for the portraits were students (27 women, 27 men) from another university, ensuring that participants would not recognize the individuals portrayed. We took 54 color portrait photographs of full faces with a neutral facial expression against a black background.

#### Behavioral self-descriptions

We initially compiled a list of single-word clearly positively and negatively valenced personality traits based on published valence ratings [22], e.g., honest, helpful, aggressive, jealous, etc. All trait adjectives were equivalently common in the German language [23]. We then composed a large set of short (from 20 to 40 words in length) self-descriptions of a behavioral episode, each of which implied one of the previously selected personality trait adjectives. We pretested the self-descriptions with 30 students, none of whom participated in the present experiment proper. The pretest students read each self-description and generated a single trait adjective that they felt characterized the described person. The present experiment then used as stimulus materials only those self-descriptions in which at least 80% of the pretest students applied the same trait adjective to it, or a close synonym, and no pretest student indicated the wrong affective valence of the trait adjective. The experimenter defined the close synonyms prior to the experiment using an online German synonym dictionary [23]. (All verbatim quotations of stimulus materials given below are of course translations into English from the original German). For example, the self-description, “I went to the grocery store yesterday. After I had paid, I realized that the cashier miscalculated the bill. I went back to the store and handed back the overpayment,” was labeled with the trait-adjective “honest” by 90% of the pretest students.

In addition, we developed 17 neutral behavioral self-descriptions that did not strongly imply a clearly positive or negative personality trait, but that were of similar length to the positive-negative trait-implying self-descriptions. An example of a neutral behavioral self-description was, “I had a virus on my computer the other day. I downloaded the newest anti-virus-program and now my computer works again.”

### Impression-Formation Task

The first procedure was the impression-formation task, which took place for all participants between 2 p.m. and 5 p.m. to control for the diurnal cycle of cortisol. Participants completed the experiment individually. The experimenter met each participant at the research laboratory and asked him or her to sit in a comfortable chair at a table, facing a computer screen. She then explained that the purpose was “to analyze emotional and physiological reactions to faces,” and she then obtained the person's written informed consent to participate. The experimenter then attached to the participant three electrodes of 45 mm diameter (Tyco Healthcare H34SG Ag/AgCl), two according to a standard lead II configuration and a third reference-electrode, for electrocardiographic (ECG) measurement. A standard pressure cuff was fixed around the left upper arm (Dinamap 1846 SX, Critikon, GE Healthcare). We placed the electrodes and the cuff at this point of the experiment (i) to emphasize the rationale about analyzing “physiological reactions,” and (ii) to allow undisturbed recording of physiological data during the tasks later on.

Thereafter, the experimenter started the computer program, asked the participant to follow the instructions on the screen, and left the room. On the screen appeared “You will see a series of portraits of people along with self-descriptions of their behavior. Please try to visualize the person engaging in the described behavior.” Participants then saw a series of 33 portrait-description pairs, each shown for 12 s, with a black screen shown for 2 s between each portrait-description pair. Each portrait was centred on the upper part of the screen, with the description centred below. Of the 33 portrait-description pairs, 8 self-descriptions implied positive traits, 8 self-descriptions implied negative traits, and the remaining 17 self-descriptions were neutral filler descriptions. The neutral self-descriptions were used to reduce the chance that participants could guess that the experiment's purpose was to study impressions of positive and negative personality traits, replicating Carlston and Skowronski's [3], [21] procedure. The first portrait-description pair, and every second remaining portrait-description pair, was neutral, and between these the positive and negative portrait-description pairs alternated. For each participant 8 positive behavioral self-descriptions and 8 negative behavioral self-descriptions were randomly drawn from a set of 16 positive and 16 negative behavioral self-descriptions. The portraits and descriptions were randomly assigned to each other, so that each participant saw different pairings of portraits and descriptions.

### Interference Task

Immediately after the impression-formation task, participants engaged in an interference task designed to reduce their ability to recall or recognize the statements they had seen during the impression-formation task, replicating the procedure used by Carlston and colleagues [3], [21]. The interference task presented to participants 40 self-descriptions of behavior in 20 pairs, with one description of each pair displayed on the screen's upper half and the other simultaneously on the screen's lower half. These 40 self-descriptions were similar in design but dissimilar in content to those in the impression-formation task. The instructions stated, “You will see pairs of self-descriptions, each describing a different individual. Please read the two self-descriptions in each pair and then indicate by a mouse click on the description itself which person you think would be more likeable” The interference task lasted 5 min for every participant.

### Pre-Stress Saliva and Blood Pressure

Immediately after the interference task, the experimenter collected a saliva sample from each participant and measured his or her blood pressure. The participant first placed a cotton swab provided in each Salivette tube in his mouth and gently chewed on it for about a min. The swab was then placed back in the Salivette tube (Sarstedt, Germany). Tubes were stored at room temperature until completion of the experimental session and were then kept at −20°C until analysis. By pressing a button, blood pressure was measured and recorded automatically. Thereafter, participants were assigned randomly to the cold pressor stress (18 females, 12 males) or the non-stressful warm water control paradigm (19 females, 11 males).

#### Stress condition: Cold-pressor test with social evaluation

The experimenter then informed participants assigned to the stress condition that (i) they would immerse their hand up to the wrist in ice water for as long as they could tolerate, (ii) the procedure will be potentially painful, and (iii) their performance would be videotaped so that the researchers could analyze the facial expressions. Again, the participants had to sign the written informed consent to the videotaping. The cold pressor (ice-water) test itself is a frequently used laboratory pain stressor [for a review see 24]. Our instructions are part of the stress procedure of the 'socially evaluated cold pressor test' described by Schwabe, Haddad, and Schachinger [25]. The putative videotaping of the procedure, the additional informed consent, no information on how long it will last, add to strengthen participants' perceptions of social evaluation, uncertainty and lack of control, which are characteristic features of a stressor.

The experimenter asked participants to immerse their right hand up to the wrist into an ice cold water bath maintained at 0–4°C, while keeping the computer screen in view to see additional instructions. A female experimenter watched the participants during the cold pressor test to maintain social surveillance. A second experimenter in a nearby control room measured participants' blood pressure after 1.5 min of the cold pressor test. After 3 min the computer screen told participants to remove their hand from the water. All participants kept their hand in the ice water for the full 3 min.

#### Control condition

The adequate control for the cold pressor test with social evaluation is the warm water test without social evaluation [25]. Here, the experimenter asked the participant to place the right hand including the wrist into a tub of warm water, which was maintained at normal body temperature (35–37° Celsius), and to keep the computer screen in view. The experimenter then left the room. There was no video camera present, no informed consent was asked. Blood pressure measurement was initiated after 1.5 min had elapsed and at 3 min the message on the computer screen told participants to remove the hand from the water. All participants kept their hand in the warm water for the full 3 min.

### Ratings of Stress, Pain, and Unpleasantness

Immediately after the participants took their hand out of the cold or warm water the computer screen prompted them to rate separately on scales ranging from 0 (“not at all”) to 100 (“very much”) in 10-point increments, first how “stressful” the previous hand immersion had been, then how “unpleasant” it had been, and then how “painful” it had been.

### Post-Stress Saliva and Blood Pressure

Directly after the ratings, the experimenter collected another saliva sample and measured the blood pressure. Then the experimenter led participants to a nearby room and collected additional saliva samples at 10, 20, 30, 45 and 60 min after the stress or control (cold or warm water) procedure was finished. After collecting the final (60-min) saliva sample, the experimenter told participants that the first day's procedure was now done, and reminded them that they needed to return the following morning to complete the experiment.

### Learning Task: Portraits and Trait-Adjective Pairs

The next morning, after the participant arrived at the laboratory, the participant first completed a computer-guided learning task in which she or he viewed 36 portraits of faces paired with a single trait adjective. Each portrait-adjective pair was displayed for 6 s, and between each pair a black screen appeared for 1 s. The computer instructed the participant, “In the next part of the experiment you will see different faces combined with words. Please try to remember each portrait-word combination. You will be asked to recall them later.”

Of the 36 portrait-trait adjective pairs, 16 portraits were “old” portraits identical with those that had been paired with positive or negative self-descriptions from the previous day's impression-formation task. These old portraits were now paired with the single trait adjective that pretest students had used to label the corresponding self-description shown the previous day (as described in the *Materials Development and Pretesting* section, above). In other words, the portraits from the initial impression formation task were now paired with the personality trait adjective implied by the self-descriptive statement that accompanied the same portrait the participant had seen in the initial impression formation task the day before. The remaining 20 portraits and each one's paired trait adjective were entirely novel; participants had not previously seen either the portrait or any trait description matching the trait adjective.

Of the 20 new portrait- trait adjective pairs, 2 appeared at the beginning of the series of 36, and 2 at the end of the series. These initial 2 and final 2 portrait- trait adjective pairs were discarded from later analyses to eliminate primacy and recency effects on learning performance. The remaining 16 novel portrait- trait adjective pairs served as control trials to measure participants' learning of new portrait- trait adjective pairs. Of these 16 novel control pairs, 8 were positive trait adjectives and 8 were negative trait adjectives, similar in valence and arousal ratings to the trait adjectives of the old portraits. Portrait-adjective pairs (except for the 2 primacy and 2 recency pairs) were presented in a random order.

### Filler Task

After completing the learning task, participants engaged in a 4 min filler task of solving a childrens' puzzle to ensure that participants' immediate short-term memory was cleared of the learning task stimuli.

### Cued Recall Procedure

Immediately after the filler task, participants' ability to recall the trait adjectives in response to the portrait cue was assessed. The 32 portraits from the learning task (i.e., those remaining after the 4 primacy-recency pairs were eliminated) were displayed on a computer screen and the computer screen instructed the participants: “Now the portraits that were paired with the words will be presented to you again one by one. Please use the keyboard to type in the word that was paired with each portrait. If you are not sure which word was paired with some of the portraits, please guess.” After finishing this cued recall procedure, participants were debriefed, and for those in the cold-water stress condition the lack of actual videotaping was explained. After making sure that no participant in either condition had further concerns about deception, pain, or any other aspect of the experiment, the experimenter thanked them for their participation, told them that the experiment was now completed, and paid them the promised 20 Euros.

### Data reduction

#### Cardiovascular data

Heart rate was derived from ECG. Beat detection and artifact control was performed offline with WinCPRS (Absolute Aliens, Oy, Turku, Finland).

#### Saliva

After thawing the saliva samples for biochemical analysis, the fraction of free cortisol in saliva was determined using a time-resolved immunoassay with fluorometric detection. The saliva of one participant in the cold pressor stress condition was missing.

#### Scoring of personality impressions

Two raters independently judged the trait-adjectives as being correctly or incorrectly recalled in the cued recall procedure. Trait adjectives were scored as correctly recalled if they were identical to the original trait adjective or a synonym, defined a priori as described above in the *Materials Development and Pretesting* section. Interrater reliability for correctly recalled adjectives was perfect, with Cohen's Kappa  = 1.00.

### Statistical Analysis

Data were analyzed by repeated measures or mixed design ANOVA as appropriate, with the alpha level set at *p*<.05. Effect sizes are reported as partial *η* ^2^.

## Results

### Manipulation Checks

To allow interpretation of our data, we had to prove that (i) some degree of impression formation had taken place and (ii) the cold pressor test had actually induced stress while the control condition had not.

#### Impression-formation

Impression-formation took place if participants recalled more trait adjectives that were previously implied (the old portrait- trait adjective pairs) than trait adjectives that were not previously implied (the new portrait- trait adjective pairs). Across all subjects and personality traits pooled, a mean of 5.32 implied (old) traits were recalled (*SD* = 2.9), whereas a mean of 3.73 non-implied (new) traits were recalled (*SD* = 2.51), a difference that was statistically significant, paired *t*(59)  = 4.68, *p*<.001, η^2^ = .27.

#### Stress induction: Stress induction

**Figure 1:**
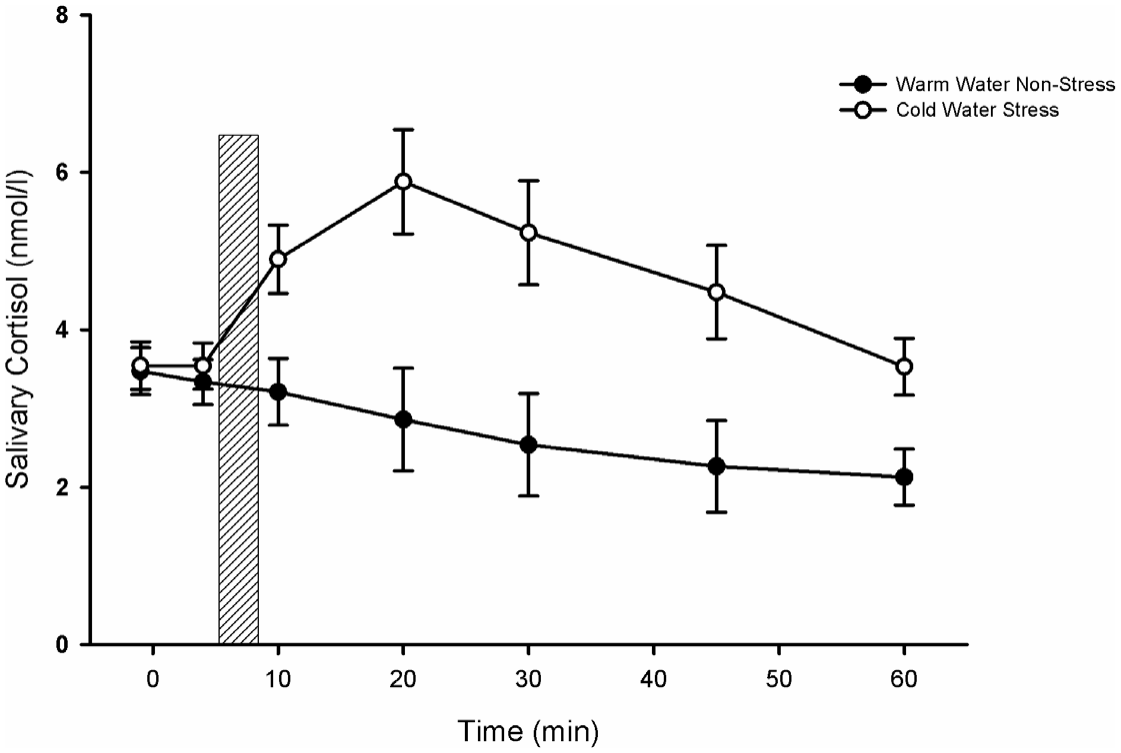
Salivary Cortisol in the Stress and the Control Group. Figure 1 presents salivary cortisol in nanomoles per liter at several time points across the experiment. The bar represents the time of the stress and the non-stress manipulation, respectively. Error bars indicate one standard error.

Subjective and physiological parameters prove that the cold pressor test is a reliable stressor. Subjective stress ratings (Table 1): Participants rated the cold pressor test significantly more stressful, painful and unpleasant (stress *F*(1, 58)  = 50.52, *p*<.001, η^2^ = .47; pain, *F*(1, 58)  = 134.78, *p*<.001, η^2^ = .70, unpleasant, *F*(1, 58)  = 109.16, *p*<.001, η^2^ = .65). Ratings of the participants in the warm water procedure were low (<10 on the scale of 1 to 100). - In response to the stressor, saliva cortisol concentrations increased significantly and returned to baseline levels after 60 min (Fig. 1; *F*(6, 342) = 7.85, *p*<.001, η^2^ = .12). Cortisol did not change in response to the warm water procedure. - Systolic and diastolic blood pressure (Table 1) increased significantly in response to the cold pressor test compared to the warm water procedure (mixed 2×3 ANOVA; systolic *F*(2, 116)  = 35.96, *p*<.001, η^2^ = .38; diastolic *F*(2, 116) = 15.35, *p*<.001, η^2^ = .21). Significant differences were observed during immersion of the hand into the cold water (systolic: *F*(1, 58)  = 29.01, *p*<.001, η^2^ = .33; diastolic: *F*(1, 58)  = 23.59, *p*<.001, η^2^ = .289), but not before or thereafter. Systolic and diastolic blood pressure remained in the normal range of healthy persons over all time points of measurement. - Heart rate (Table 1) was not significantly affected by the cold pressor procedure (*F*(2, 116) = 2.49, *p* = .087).

**Table 1:** Heart rate (beats per minute), systolic and diastolic blood pressure (mmHg) before (pre), during and after (post) hand immersion in warm or cold water as well as subjective stress ratings in the two treatment group (cold pressor test vs. warm water test).

		Warm Water Test		Cold Pressor Test	
Heart Rate					
	Pre	76.32±2.58		80.77±2.81	
	During	72.97±3.2		77.38±3.48	
	Post	76.52±2.16		74.65±2.36	
Systolic Blood Pressure					
	Pre	117.13±2.39		119.08±2.61	
	During	114.00±2.45		**134.73**±**2.67** *	
	Post	115.87±2.71		122.77±2.96	
Diastolic Blood Pressure					
	Pre	71.58±1.85		71.08±2.02	
	During	69.71±1.79		**83.07**±**1.96** *	
	Post	68.77±1.77		73.31±1.93	
Subjective Stress Ratings					
	Unpleasant	7.01±3.11		**60.39**±**3.40** *	
	Stressful	8.71±3.46		**46.15**±**3.77** *	
	Painful	1.61±2.92		**62.69**±**3.19** *	

*p<.001 compared to warm water test. Data represent M ± SEM.

### Effects of Stress on Impression Formation

There was neither a gender effect (*F*(1, 56)  = .000, p = .984) nor a gender-stress interaction in memory for the new and the old personality traits (*F*(1, 56)  = 1.11, p = .297). Therefore data of male and female participants were pooled for further analysis.

We expected that stress will increase the retention of first personality impressions. As indexed above the difference between memory for previously implied personality (old) trait adjectives and not-implied (new) trait adjectives serves as an index for impression formation.

#### Stress effects on first trait impressions for personality traits

We conducted a two (learning trial type: implied (old) vs. not-implied (new)) x two (trait adjective valence: positive vs. negative) x two (stress condition: stress group vs. control group) mixed design ANOVA with memory for personality traits as dependent variable. There was no interaction between stress condition and learning trial type (*F*(1, 58)  = .204, p = .723). Participants in the stress and in the control group remembered about the same amount of implied (old) trait adjectives and not-implied (new) trait adjectives. However, there was a significant three way interaction between learning trial type, trait adjective valence and stress condition (*F*(1, 58)  = 9.83, p<.01, η^2^ = .15). Simple main effects showed that the stress group shows a significant higher learning performance for positive implied (old) trait adjectives compared to positive not implied (new) trait adjectives (*T*(29)  = 5.311, p<.001). This finding was not present in the control group: Participants in the control group did not show a significant higher learning performance for positive implied trait adjectives than positive not-implied trait adjectives (*T*(29)  = 1.45, p = .16). For negative pictures, the opposite picture emerged: Participants in the stress group did not show a better learning performance for negative implied trait adjectives compared to negative not-implied trait adjectives (*T*(29)  = −.720, p = .477). In the control group on the other hand, there was a higher learning performance for negative implied traits compared to negative not-implied trait adjectives (*T*(29)  = −3.075, p<.05).

**Figure 2:**
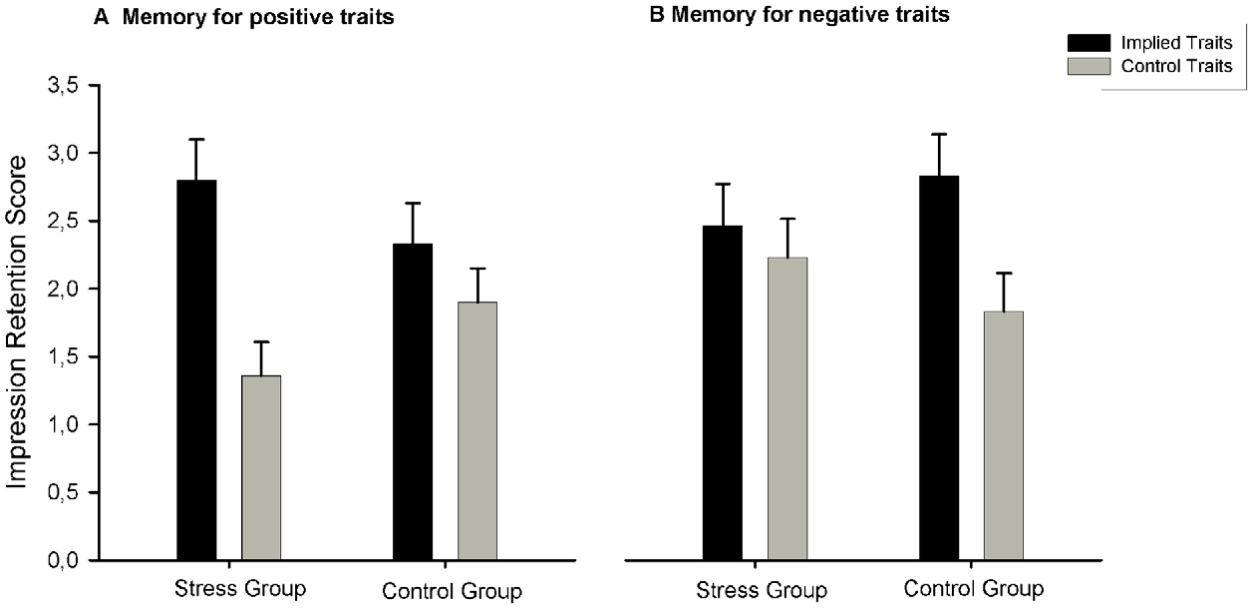
Impression retention in the Stress and the Control Group. Figure 2 presents the impression retention for positive (Figure 2 a) and negative traits (Figure 2 b) in the two experimental groups (stress vs. non-stress). Error bars indicate one standard error.

Participants in the stress group also showed a higher learning performance for negative control traits compared to positive control traits (*T*(29)  = −3.37, p<.01) Figure 2 illustrates these findings.

We furthermore conducted correlation analysis in order to analyze whether the single indicators of stress reactivity correlated with memory for positive and negative implied trait adjectives. There were no significant correlations between the single indicators of stress reactivity (Blood Pressure, Heart Rate, Subjective Ratings, Cortisol Increase) and memory for implied traits (positive and negative) within the stress and control group (all r's <.20, n.s.).

## Discussion

The present findings indicate that stress **after** acquiring information about a previously unknown person, strengthens the memory of the newly formed impressions for positive personality traits. Both groups showed significant impression formation. However, while the control group showed higher impression retention for negative trait adjectives compared to positive trait adjectives, the stress group showed higher impression retention for positive trait adjectives compared to negative trait adjectives. That the experimental stressor was actually stressful was verified both by subjective measures of stress, pain, and unpleasantness, but importantly also by physiological measures of stress including cortisol and systolic and diastolic blood pressure. These findings replicated those of Carlston and Skowronski [3] in a computerized version of their personality impression-formation paradigm, and importantly extended it by showing that such memory for earlier brief personality impressions is modulated by post-learning stress.

That stress modulates retention of first personality impressions extends the knowledge about the effects of stress on human emotion and cognition to the field of social cognition. Research has repeatedly shown that stress affects emotional memory consolidation. Cognitive effects of stress are predominantly reported for declarative and emotional memories related to hippocampus and amygdala function. Facilitation of memory consolidation was found especially for emotionally arousing material with negative, aversive [26], [27], but also with positive, appetitive affective valence [28], [29], [30]. However, that stress affects retention of personality impressions was to date unknown. The effects of stress on retention of earlier impressions are especially interesting, because impression formation and retention is a special form of learning that relies on a distinct set of cognitive processes which distinguishes it from other kinds of learning [11], [13], [14]. The present research showed that stress also affects impression formation, one of the distinct cognitive processes underlying social behavior.

Impression formation occurs incidentally, and represents a form of rather implicit learning without explicit remembering on how the impressions were formed. While the effects of stress on explicit, declarative hippocampus dependent memory have been replicated many times, effects of stress on implicit memory processes that are independent of the hippocampus are less clear. Thus, our findings represent an example that post-learning stress may also affect social relevant implicit memory processes.

In the control group we found better memory retention for negative impressions compared to positive impressions. Although there are no studies directly comparing memory for positive and negative trait adjective - face associations after an impression formation task, research showed that negative personality traits draw more attention and are weighed to be more important than positive personality traits [31], [32]. Thus, these findings are in line with the general negativity bias in memory.

However, in the stress group, we found a selective enhancement of stress on memory for positive personality traits. While the control group showed more impression retention for negative personality traits than for positive personality traits, the stress group showed more impression retention for positive personality traits than for negative personality traits.

Impression-formation is known to differ across individuals and situations. Currently fearful people, for example, tend to see more anger in neutral target persons who belong to an out-group than do non-fearful people [33]. This seems adaptive from an evolutionary point of view: In a moment of vulnerability (fearfulness) it seems reasonable to distrust strangers. However, research has also shown that people threatened with social exclusion express greater interest in making new friends, and retain more positive first impressions of novel interaction partners, than do those not threatened with social exclusion [34]. Such behavior also seems adaptive: While the fearful person distrusts strangers, the socially excluded person is, as a social being, likely to be seeking social affiliation and therefore would tend to find positive impressions of strangers more memorable.

Forming quick and accurate impressions of others and to remember these is obviously adaptive: Previous research indicated stress to enhance consolidation of information gained prior to the stressor. As such, stress strengthens memories of potential significance for the organism's survival, e.g. to deal better and earlier with the stressor the next time. Facing stress, injury or sickness, it will prove beneficial to identify potential enemies, aggressors and non-helpers. It seems quiet save to speculate that, concerning larger and complex group constellations, as present in ancient and modern human life, it is more cost-beneficial to remember a single helper and supporter ready to approach than to remember all the potential aggressors necessary to avoid. Moreover, remembering people with positive personality traits who were met in proximity of stress (e.g. shortly before the stress onset) may have two distinct advantages: First, especially those people met in proximity of the stressor may have acquired knowledge and skills to survive and cope with the very same stressor. Thus, knowing such people may selectively enhance the chance to receive crucial information in order to deal with the stressor. Second, people that behaved empathic and friendly before the stressful situation had emerged, may be the true altruistic helpers, in contrast to temporizers who might act helpful after the stressor, because they expect some immediate advantage of it.

Such stress-mediated effects favouring the recognition of potential helpers fit in well with a new theory on the biobehavioral stress response that has been proposed by Taylor and colleagues [35]. They found the female stress response to be poorly described by “fight and flight” responsiveness but rather by a pattern they term “tend and befriend” strategy. They suggest that females respond to stress by befriending, namely affiliating with other people, to reduce risk in a stressful situation. The physiological mechanisms mediating this response strategy may well link to the female nurturing and bonding hormone oxytocin, which is released in response to stressful events and attenuates the endocrine and autonomic stress response [36], [37]. Oxytocin enhances prosocial behavior and affiliation, but importantly, such effects may be extended to social relevant memory, and to both sexes. For example, Guastella and colleagues showed that a single dose of oxytocin enhances the encoding of positive social memories in male participants [38]. Thus, the enhanced memory for positive impressions found in this study may be related to stress oxytocin release. Since it was not possible to measure oxytocin levels in the currently designed study, future studies should incorporate measurements to address this issue in an optimally sex-balanced sample.

At first glance, in the stress group the better memory for positive personality traits seems to be at the expense of memory for negative personality traits. We did not find significant impression formation for negative personality traits in the stress group. However, participants in the stress group showed a rather high memory for negative control traits compared to positive control traits. Therefore the non-significant results for the effects of stress on negative personality traits, might be due to the high learning performance for negative control traits, and implicating some kind of a ceiling effect. The high level of learning performance for negative control traits may be induced by currently unexplained context factors, such as merely returning to the very same experiment, and has mathematically an effect on the relatively low difference between memory for implied and for control traits in the cold pressor stress group. Therefore, we cannot safely conclude that the memory enhancement for positive personality traits is at the expense of memory for negative personality traits.

The present finding extend the knowledge of the influence of stress on memory processes, and suggests that stress also influences the memory of our spontaneous impressions of strangers' personality traits. In evolution, a fundamental psychological goal is the detection of other people who might harm us. Our study seems to indicate that in a physically and socially stressful situation we show a better memory for potential helpers than in a non-stressful situation. Of course, this interpretation requires further exploration. The stress-induced strengthening of the memory of other peoples' positive personality traits might represent an important social element for the formation of alliances in stressful circumstances.
